# Improvement of Thermotolerance of *Zymomonas mobilis* by Genes for Reactive Oxygen Species-Scavenging Enzymes and Heat Shock Proteins

**DOI:** 10.3389/fmicb.2019.03073

**Published:** 2020-01-30

**Authors:** Sakunda Anggarini, Masayuki Murata, Keisuke Kido, Tomoyuki Kosaka, Kaewta Sootsuwan, Pornthap Thanonkeo, Mamoru Yamada

**Affiliations:** ^1^Division of Life Science, Graduate School of Sciences and Technology for Innovation, Yamaguchi University, Ube, Japan; ^2^Department of Biological Chemistry, Faculty of Agriculture, Yamaguchi University, Yamaguchi, Japan; ^3^Research Center for Thermotolerant Microbial Resources, Yamaguchi University, Yamaguchi, Japan; ^4^Faculty of Agro-Industrial Technology, Rajamangala University of Technology Isan, Kalasin, Thailand; ^5^Department of Biotechnology, Faculty of Technology, Khon Kaen University, Khon Kaen, Thailand

**Keywords:** critical high temperature, thermotolerance, reactive oxygen species-scavenging enzyme, heat shock protein, *Zymomonas mobilis*

## Abstract

Thermotolerant genes, which are essential for survival at a high temperature, have been identified in three mesophilic microbes, including *Zymomonas mobilis*. Contrary to expectation, they include only a few genes for reactive oxygen species (ROS)-scavenging enzymes and heat shock proteins, which are assumed to play key roles at a critical high temperature (CHT) as an upper limit of survival. We thus examined the effects of increased expression of these genes on the cell growth of *Z. mobilis* strains at its CHT. When overexpressed, most of the genes increased the CHT by about one degree, and some of them enhanced tolerance against acetic acid. These findings suggest that ROS-damaged molecules or unfolded proteins that prevent cell growth are accumulated in cells at the CHT.

## Introduction

Microorganisms intrinsically have an upper temperature limit for survival called a critical high temperature (CHT) (Matsushita et al., 2015; Kosaka et al., 2019). Genome-wide analysis of three mesophiles, *Escherichia coli*, *Acetobacter tropicalis*, and *Zymomonas mobilis*, by screening thermosensitive mutants either with a single-knockout mutant library or with a transposon-inserted mutant library has revealed that about 1.5% of genomic genes, called thermotolerant genes, are responsible for cell survival at a CHT (Charoensuk et al., 2017; Murata et al., 2018), but there is no sufficient information to conclude that a mesophile with a larger number of genomic genes has a larger number of thermotolerant genes and thus tends to be more temperature-resistant. Other factors including gene expression of key proteins may also contribute to the degree of thermotolerance. Thermotolerant genes are categorized into nine groups, including genes for metabolism, membrane stabilization, transporter, DNA repair, tRNA modification, protein quality control, translation control, cell division, and transcriptional regulation (Murata et al., 2011, 2018; Soemphol et al., 2011; Charoensuk et al., 2017), which are mostly related to fundamental activities of cells. The CHT differs to some extent from strain to strain even in the same species such that relatively thermotolerant strains can be isolated in tropical areas, assuming that thermotolerant strains have adapted to the environmental temperature. The thermotolerance of mesophiles, *E. coli* W3110, *Z. mobilis* CP4, and *Z. mobilis* TISTR 548, has been improved by *in vivo* thermal adaptation, suggesting that they have a genomic capacity for adaptation to higher temperature environments (Kosaka et al., 2019). The capacity, however, is limited to 2–3°, and the variation of thermal adaptation is also restricted. The change of only a few degrees is physiologically important, which was determined by an accurate method, called a two-step cultivation assay that eliminates the effects of start temperature and can distinguish CHT differences between two different strains of the same species (Kosaka et al., 2019).

The thermotolerance of microbes is remarkably beneficial for stable fermentation. Ethanologenic microbes, for example, are exposed to heat stress in the ethanol fermentation process (Attfield, 1997; Wang et al., 2007) due to its exothermic reaction (van Uden and da Craz Duarte, 1981; Ghose and Bandyopadhyay, 1982). Heat stress has a negative impact on their growth or viability (Basso et al., 2008; Babiker et al., 2010) and prevents their fermentation ability. These negative effects are enhanced in the presence of other factors, including a low pH, a high ethanol concentration, or a high osmolarity (Piper, 1995; Carmelo et al., 1998; Ciani et al., 2006; Coleman et al., 2007; Gibson et al., 2007; Pizarro et al., 2007). Thus, heat stress should be avoided for stable and effective fermentation by chilling fermentation reactors. Thermotolerant microbes enable high-temperature fermentation (HTF) to be performed, thus reducing the costs for chilling reactors in the bioconversion process of biomass to fuels or chemicals and for biomass hydrolysis in simultaneous saccharification fermentation and thus preventing contamination of other microbes (Murata et al., 2015).

A higher temperature results in accumulation of more oxidative stress in *E. coli* (Noor et al., 2009), and oxidative stress is involved in heat-induced cell death as has been shown for *Saccharomyces cerevisiae* (Davidson et al., 1996), being consistent with findings that overexpression of genes for catalase and superoxide dismutase is able to increase the degree of thermotolerance (Nantapong et al., 2019) and that the thermotolerance increases under anaerobic conditions (Davidson et al., 1996; Davidson and Schiestl, 2001). It is thus assumed that the impact of the CHT causes intracellular oxidative stress to elicit harmful effects on cells as a secondary stress. However, only one gene and no gene for reactive oxygen species (ROS)-scavenging enzymes (RSEs) was found as a thermotolerant gene in *A. tropicalis* and *Z. mobilis*, respectively (Soemphol et al., 2011; Charoensuk et al., 2017). The CHT would also cause damage of proteins to be unfolded or denatured. Surprisingly, no genes for general heat shock proteins (HSPs), except for *degP*, *dnaK*, and *dnaJ* in *E. coli* (Murata et al., 2011, 2018), *degP* in *A. tropicalis* (Soemphol et al., 2011), and *degP* in *Z. mobilis* (Charoensuk et al., 2017), have been identified as thermotolerant genes.

In this study, we thus examined the effects of increased expression of genes for RSEs and HSPs on cell survival at the CHT in *Z. mobilis*. Increased expression of most of the genes tested raised the CHT and reduced ROS compared to the controls. These findings together with previous findings suggest that the CHT is determined by functional contributions of several factors that prevent the accumulation of damaged macromolecules in cells in addition to fundamental activities by thermotolerant genes identified previously.

## Materials and Methods

### Materials

Oligonucleotide primers were purchased from Greiner Bio-One (Japan). A DNA purification kit, gel extraction kit, and one-step RT-PCR kit were from Qiagen (Japan). Restriction enzymes were from Biolabs (Japan) and Takara (Japan). PrimeSTAR DNA polymerase and an In-Fusion HD cloning kit were purchased from Takara (Japan). *E. coli* DH5α was from Toyobo (Japan). Yeast extract, peptone, tryptone, and agar were from Nacalai Tesque (Japan). Glucose and NaCl were from Sigma-Aldrich (United States). Chloramphenicol was from Boehringer Mannheim GmbH (Germany). Other chemicals used in this study were of analytical grade.

### Bacterial Strains, Media, and Culture Conditions

Plasmids used in this study are listed in Supplementary Table S1. *Z. mobilis* TISTR 548 (Sootsuwan et al., 2007) was cultured in YPD medium [0.5% (w/v) yeast extract, 0.3% (w/v) peptone, and 3% (w/v) glucose]. Recombinant plasmids with targeted genes were introduced into *E. coli* DH5α (Toyobo, Japan) cells, and the cells were incubated in LB medium (1% tryptone, 0.5% yeast extract, and 0.5% NaCl) for 2 h and spread onto agar plates containing 1.5% agar. When pZA22 (Misawa et al., 1986) or its derivatives were introduced, agar plates were supplemented with chloramphenicol (50 μg ml^–1^). A log-phase culture, 0.5–1.0 at OD_550_, that had been prepared at 30°C was inoculated into a liquid medium, and cultivation was conducted under a static condition. Bacterial growth was monitored by measuring the optical density of the culture on a spectrophotometer (HITACHI, U-200) at OD_550_. Determination of the CHT was carried out by the two-step cultivation assay as described previously (Kosaka et al., 2019). For measuring the cell length, 12-h cultivated cells in the first culture of the two-step cultivation were collected by low-speed centrifugation, washed, and resuspended in a saline solution. The morphology of the resuspended cells was then observed under a microscope (Eclipse E600, Nikon, Japan) with 400 × magnification, and images of the same sample were taken three to five times. The lengths of approximately 100 cells were manually measured.

### Construction of Expression Plasmids of Genes for RSEs and HSPs

Conventional recombinant DNA techniques were applied (Sambrook et al., 1989). For increased expression of genes for RSEs and HSPs in *Z. mobilis* TISTR548, operon fusion genes of the *pdc* promoter and each of these genes were constructed and incorporated into pZA22 (Misawa et al., 1986) as an expression vector for *Z. mobilis*. The *pdc* promoter fragment including the Shine–Dalgarno sequence of *pdc* (514 bp) and each gene fragment from its initiation codon to 20 bp downstream from its stop codon were amplified by PCR using the genomic DNA of TISTR548 as a template. pZA22 was linearized by PCR. The primers (Supplementary Table S2) used for PCR were designed according to the In-Fusion HD cloning method. The PCR fragments were purified using a QIAquick gel extraction kit and connected by an In-Fusion HD cloning kit. The constructed plasmids were confirmed by PCR and restriction mapping.

### RT-PCR

To examine the degree of increased expression of targeted genes, RT-PCR was performed as described previously (Murata et al., 2011, 2018). Precultured cells were inoculated and cultivated in YPD medium containing chloramphenicol at 30°C for 12 h. The cells were then harvested, and total RNA was prepared by the hot phenol method (Aiba et al., 1981). RT-PCR was performed using a One-Step RNA PCR Kit with two specific primers for each gene (Supplementary Table S3) and 0.5 μg of total RNA according to the protocol from the kit supplier. After RT reaction at 50°C for 30 min, PCR consisting of denaturing at 94°C for 30 s, annealing at 50°C for 30 s, and extension at 72°C for 1 min was carried out. The PCR products after 10, 15, 20, and 25 cycles for each gene were taken and analyzed by 1.2% agarose gel electrophoresis, followed by staining with ethidium bromide. The intensity of bands of RT-PCR products was quantitatively determined using ImageJ. The linearity of the amplification was observed up to the 25th or 35th cycle. Under our conditions, the RT-PCR was able to specifically detect mRNA because no band was observed when reverse transcriptase was omitted.

### Determination of ROS

The level of intracellular ROS was determined using a fluorescence probe, 2′,7′-dichlorofluorescin diacetate (H2DCFDA) (Pérez-Gallardo et al., 2013), as described previously (Kosaka et al., 2019). Cells that were grown for 12 h at 38°C in the first culture of the two-step cultivation assay were mixed with H2DCFDA at the final concentration of 5 μM, incubated for 30 min, and collected as a pellet by centrifugation (14,000 rpm) for 1 min. The pellet was then washed with saline solution, resuspended in 10 mM potassium phosphate buffer (pH 7.0), and disrupted by sonic oscillation (Cosmo Bio Japan). The fluorescence was measured using a POWERSCAN HT microplate reader (DS Pharma Biomedical Osaka Japan) with excitation at 485 nm and emission at 582 nm. Emission values were normalized by protein concentration, which was determined by the Lowry method (Dulley and Grieve, 1975).

### Observation of Stress Resistance

To examine the effects of increased expression of genes on resistance to stresses other than high temperatures, cells were precultured at 30°C until a mid-log phase, diluted (10^0^–10^4^), and spotted on YPD agar plates supplemented with 6–2% glucose, 3–5% ethanol, or 0.03–0.3% acetic acid. The plates were then incubated at 30°C for 48 h. To examine the effect of H_2_O_2_ on cell growth, precultured cells were inoculated and cultivated at 30°C in YPD liquid medium containing H_2_O_2_ at a final concentration of 0.1 mM. The optical density of the culture at OD_550_ was then measured at 12 h. All experiments were triplicated.

## Results

### Effects of Increased Expression of Genes for RSEs and HSPs on Growth at the CHT

**FIGURE 1:**
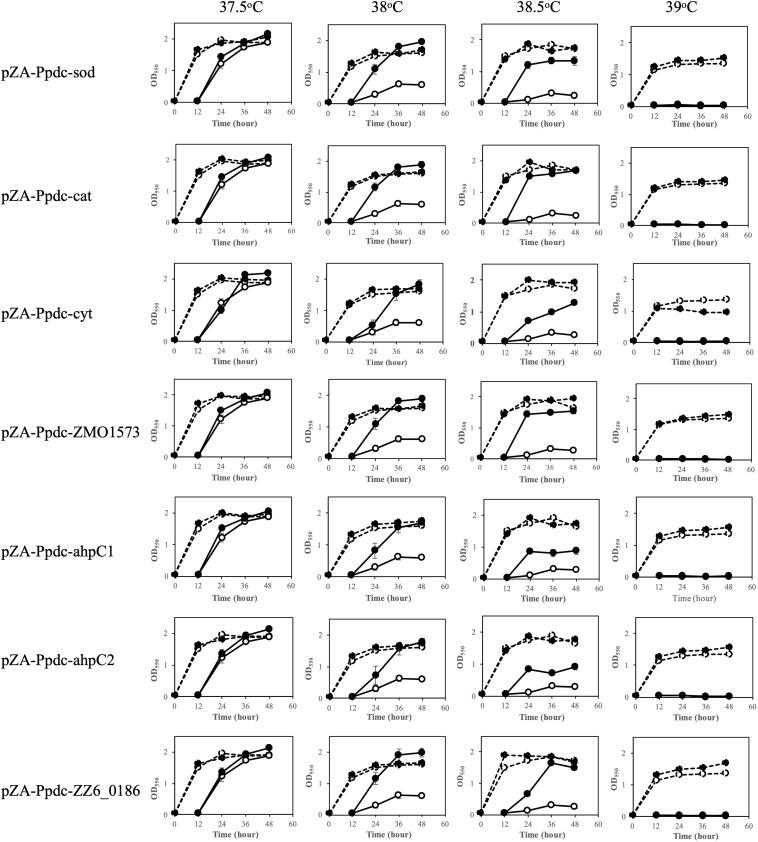
Effects of increased expression of genes for reactive oxygen species (ROS)-scavenging enzymes (RSEs) on growth at the critical high temperature (CHT). Growth of cells harboring a recombinant plasmid with a gene for one of the RSEs (closed circles) or an empty plasmid (open circles) was examined by two-step cultivation at 37.5–39°C in YPD medium containing chloramphenicol. After 12 h in the first cultivation (dotted lines) at each temperature, an aliquot of the culture was transferred to a fresh medium, and the second cultivation was carried out (straight lines). At the times indicated, cell density was estimated by measuring OD_550_. Bars represent ± SD for three independent experiments.

To examine the contribution of RSEs and HSPs to the survival of *Z. mobilis* at the CHT and to the improvement of the CHT, the genes coding for these enzymes and proteins (Table 1) were individually cloned into pZA22 under the control of the *pdc* promoter, which is a relatively strong promoter derived from *Z. mobilis*, and the effects of expression of these genes were evaluated by the two-step cultivation assay, which enables determination of the CHT of mesophiles (Kosaka et al., 2019). The expression of cloned genes was confirmed by RT-PCR, indicating 1.4-to 4.0-fold increases compared to the expression of intrinsic genes in the genome (Supplementary Figure S1). Two-step cultivation assays for RSE genes were conducted at 37.5, 38, 38.5, and 39°C (Figure 1). At 38°C, all transformants with pZA-Ppdc-sod, pZA-Ppdc-cat, pZA-Ppdc-cyt, pZA-Ppdc-ahpC1, pZA-Ppdc-ahpC2, or pZA-Ppdc-ZMO1573, which bear *sod*, *cat*, *cyt*, *ahpC1*, *ahpC2* [corresponding to *ahpC* (Charoensuk et al., 2011)], or *ZMO1573*, respectively, exhibited growth in the second culture, but the transformant with an empty vector as a control hardly grew. Among the genes tested, *sod*, *cat*, *ZMO1573*, and *ZZ6-0186* had relatively stronger effects on cell growth, especially after 36 h. At 38.5°C, transformants with pZA-Ppdc-sod, pZA-Ppdc-cat, pZA-Ppdc-ZMO1573, or pZA-Ppdc-ZZ6-0186 showed clearly stronger growth than those with pZA-Ppdc-cyt, pZA-Ppdc-ahpC1, pZA-Ppdc-ahpC2, or pZA-Ppdc-ZMO1573 in the second culture. The two-step cultivation data indicated that the CHTs of transformants with pZA-Ppdc-sod, pZA-Ppdc-cat, pZA-Ppdc-ZMO1573, or pZA-Ppdc-ZZ6-0186 were 38.5°C and that the CHTs of the remaining transformants were between 38 and 38.5°C. The improvement of CHT in the former group was 1°C compared to the CHT, 37.5°C, of the transformant with an empty vector and that of the latter was more than 0.5°C and less than 1°C. These findings suggest that all of the tested genes are able to improve the CHT of *Z. mobilis* TISTR 548 and that *sod*, *cat*, and *ZMO1573* are more effective.

**TABLE 1:** Genes for reactive oxygen species (ROS)-scavenging enzymes (RSEs) and heat shock proteins (HSPs) that were examined in this study.

**Genes**		**Query coverage^a^ (%)**	**Identity^a^ (%)**	**Function**	**References**
***Z. mobilis***	***E. coli***				
**RSE’s genes**					
*Sod*	*sodB*	98	52	Superoxide dismutase, Fe-Mn family	Davidson et al., 1996
*Cat*	*katE*	51	49	Catalase	Davidson et al., 1996
*Cyt*	*yhjA*	96	44	Predicted cytochrome C peroxidase	Charoensuk et al., 2011
*ZZ6 1529*	*yfeX*	89	36	Dyp-type peroxidase family	Matsushita et al., 2015
*ahpCl*	Not applicable			AhpC/TSA family protein	La Carbona et al., 2009
*ahpC2*	*ahpC*	88	38	Alkyl hydroperoxide reductase subunit C	Seaver and Imlay, 2001
*ZZ6_0186*	*trxB*	95	55	Thioredoxin reductase	Williams, 1995; Arnér and Holmgren, 2000
**HSP’s genes**					
*degP*	*degP*	92	34	Serine protease Do	Jones et al., 2002; Murata et al., 2011; Charoensuk et al., 2017
*dnaK*	*dnaK*	99	66	Molecular chaperone DnaK	Tomoyasu et al., 1998
*dnaJ*	*dnaJ*	98	52	Molecular chaperone DnaJ	Tomoyasu et al., 1998
*groEL*	*groEL*	96	67	Chaperonin GroEL	Tomoyasu et al., 1998
*groES*	*groES*	97	52	Chaperonin GroES	Tomoyasu et al., 1998
*hslU*	*hslU*	98	53	ATP-dependent HslUV protease ATP-binding subunit HslU	Seol et al., 1997; Yoo et al., 1998
*ibpA*	*ibpA*	99	50	Heat shock protein Hsp20	Kitagawa et al., 2002; Kuczyńska-Wiśnik et al., 2002
*clpA*	*clpA*	97	59	ATP-dependent Clp protease ATP-binding subunit ClpA	Katayama et al., 1988; Kress et al., 2009
*clpB*	*clpB*	99	58	ATP-dependent Clp protease ATP-binding subunit ClpB	Barnett et al., 2000; Mogk et al., 2015;
					Kedzierska et al., 2003
*clpS*	*clpS*	92	55	ATP-dependent Clp protease adaptor protein ClpS	Dougan et al., 2002; Román-Hernández et al., 2011
*ZZ6 0844*	*hslO*	91	33	Molecular chaperone Hsp33	Jakob et al., 1999

*^*a*^Query coverage and identity of each gene were compared with *Z. mobilis* TISTR548 and *E. coli* W3110.*

**FIGURE 2:**
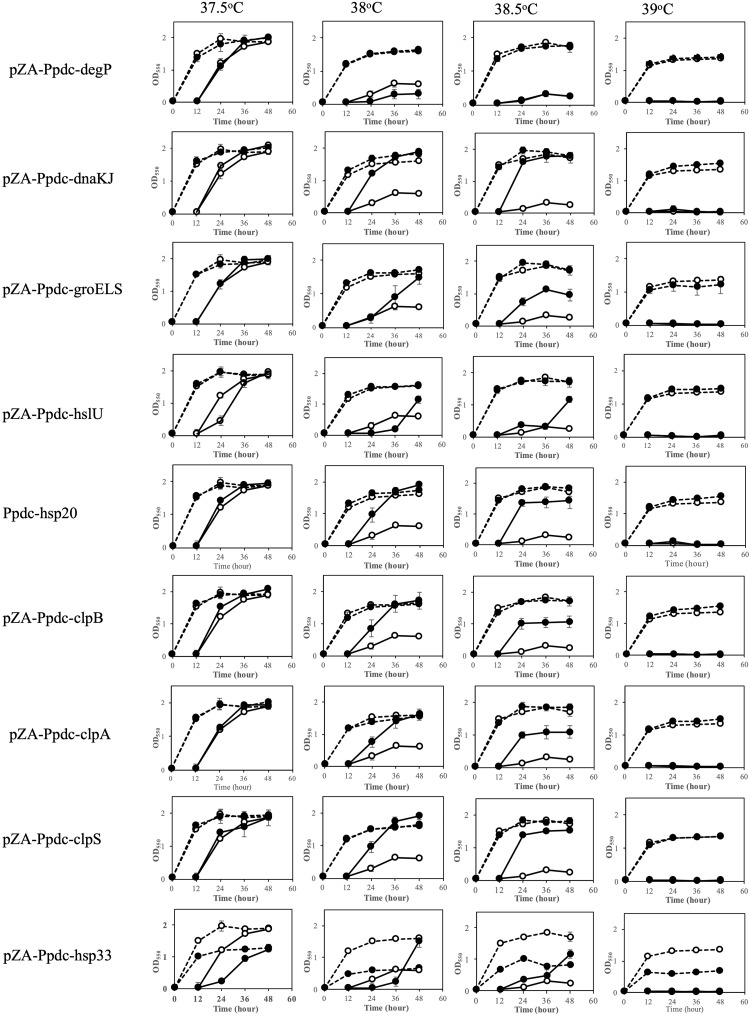
Effects of increased expression of genes for heat shock proteins (HSPs) on growth at the critical high temperature (CHT). Growth of cells harboring a recombinant plasmid with a gene for one of the HSPs (closed circles) or an empty plasmid (open circles) was examined by two-step cultivation at 37.5–39°C in YPD medium containing chloramphenicol as shown in Figure 1. Bars represent ± SD for three independent experiments.

The effects of increased expression of genes for HSPs were also examined by two-step cultivation in the range of 37.5–39°C, in which transformants with pZA-Ppdc-degP, pZA-Ppdc-dnaKJ, pZA-Ppdc-groELS, pZA-Ppdc-hsp20, pZA-Ppdc-hisU, pZA-Ppdc-clpB, or pZA-Ppdc-clpPX, which bear *degP*, *dnaKJ*, *groELS*, *hsp20*, *hisU*, *clpB*, or *clpPX*, respectively, were used (Figure 2). At 38°C, all transformants except for a transformant with pZA-Ppdc-degP exhibited higher growth in the second culture than the growth of the transformant with an empty vector. Of these, transformants with pZA-Ppdc-dnaKJ, pZA-Ppdc-Hsp20, pZA-Ppdc-clpB, pZA-Ppdc-clpA, or pZA-Ppdc-clpS exhibited higher turbidity than that of the other transformants. On the other hand, cells containing pZA-Ppdc-degP showed only slightly higher turbidity than that of cells harboring an empty vector. At 38.5°C, in comparison with cells harboring an empty vector, cells harboring pZA-Ppdc-dnaKJ, pZA-Ppdc-Hsp20, or pZA-Ppdc-clpS showed relatively high turbidity in the second culture, followed by cells harboring pZA-Ppdc-groELS, pZA-Ppdc-clpB, or pZA-Ppdc-clpA. In the case of transformants with pZA-Ppdc-hslU or pZA-Ppdc-hsp33, their growth in the second culture was greatly repressed until 36 h compared to the control, suggesting that overexpression of *hslU* or *hsp33* somehow hampers the cell growth. Notably, the transformant with pZA-Ppdc-hsp33 showed lower turbidity even in the first culture. These findings suggested that increased expression of all of the genes tested except for *degP*, *hslU*, and *hsp33* can improve the thermotolerance of *Z. mobilis* TISTR 548 and that *dnaKJ*, *hsp20*, and *clpS* can up-shift the CHT by 1°C and *groELS*, *clpB*, and *clpA* can up-shift the CHT by 0.5–1°C.

### Effects of Increased Expression of Genes for RSEs and HSPs on Accumulation of ROS at the CHT

**FIGURE 3:**
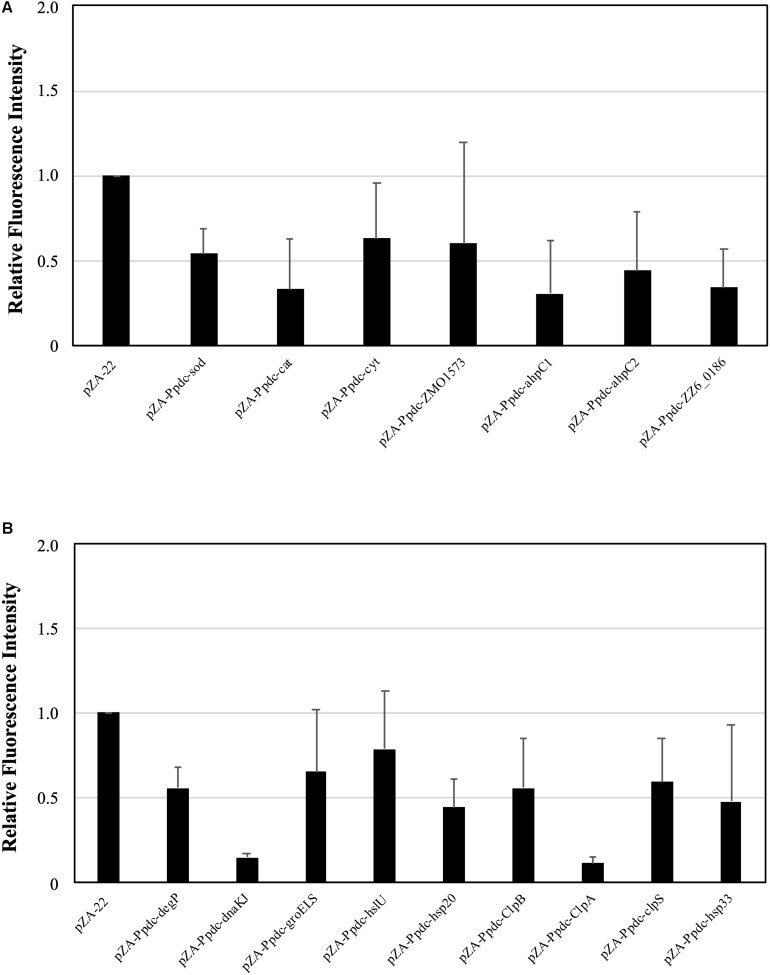
Effects of increased expression of genes for reactive oxygen species (ROS)-scavenging enzymes (RSEs) **(A)** and heat shock proteins (HSPs) **(B)** on accumulation of ROS at the critical high temperature (CHT). Cells were grown at 38°C in YPD medium containing chloramphenicol. Using the culture at 10 h, ROS were detected with H_2_DCFDA, and the fluorescent intensity reflects the level of accumulation of ROS. Bars represent ± SD for three independent experiments.

When cells are exposed to a temperature close to the CHT, the level of intracellular ROS increases (Kosaka et al., 2019). Since enhancement of the expression of genes for RSEs and HSPs caused an up-shift of the CHT, it was assumed that increased expression of these genes prevented the accumulation of ROS. We thus examined the assumption at 38°C after 12 h in the first culture (Figure 3) because accumulation of ROS in the first culture has been reported to have an impact on the growth of cells in the second culture (Kosaka et al., 2019). As a result, the level of ROS of all of the transformants with a plasmid bearing one of the genes for RSEs and HSPs was lower than that of the control. However, the ROS level at the mid-log phase in the first culture was not always consistent with the level of improvement in the CHT because transformants with pZA-Ppdc-dnaKJ and pZA-Ppdc-clpA showed the lowest level of ROS but improved the CHT by 1°C and less than 1°C, respectively. This inconsistency may be due to other contribution of the genes, especially HSP genes, to the up-shift of the CHT in addition to the reduction of ROS.

### Effects of Increased Expression of Genes for RSEs and HSPs on Cell Morphology at the CHT

**FIGURE 4:**
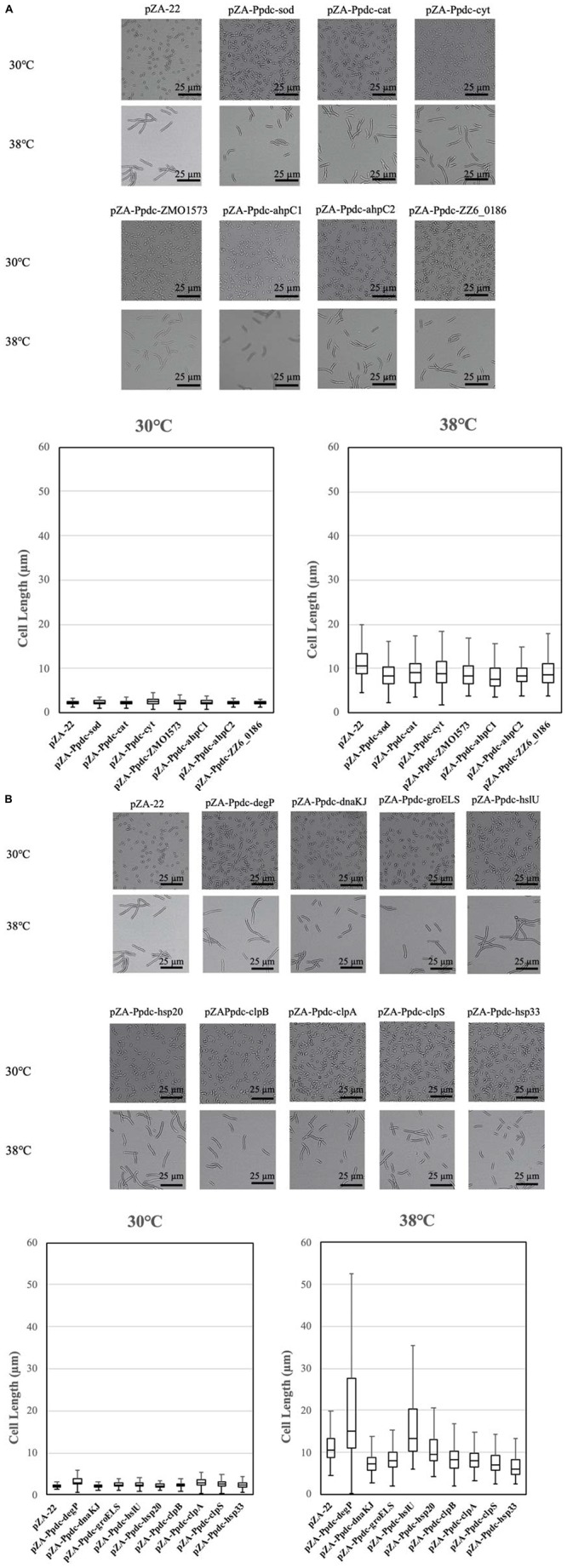
Effects of increased expression of genes for reactive oxygen species (ROS)-scavenging enzymes (RSEs) **(A)** and heat shock proteins (HSPs) **(B)** on cell morphology at the critical high temperature (CHT). Cells were grown at 38°C in YPD medium containing chloramphenicol. Using the culture at 12 h, cell morphology was observed, and lengths of 100 cells were measured.

Cells become elongated when exposed to a temperature close to the CHT (Kosaka et al., 2019). The morphological change may be due to accumulation of stress including stress caused by ROS in cells, which presumably influences cell division. As mentioned above, the enhanced expression of genes for RSEs and HSPs reduced the intracellular level of ROS. We thus assumed that their enhanced expression prevents the morphological change. The assumption was examined by measurement of cell length at 38°C after 12 h in the first culture (Figure 4). As expected, all transformants with a plasmid bearing one of the genes for RSEs were shorter in cell length than the transformant with an empty vector, being consistent with the impact of the genes on cell growth at the CHT. While the transformants with *dnaKJ*, *groELS*, *clpB*, *clpA*, *clpS*, and *hsp33* for HSPs were relatively short in cell length, the transformant with *hsp20* showed no change, and the transformant with *degP* or *hslU* were much larger. Considering the lower levels of ROS in the transformants, increased expression of the latter three genes may somehow hamper cell division.

### Effects of Increased Expression of Genes for RSEs and HSPs on Growth Under Conditions of Stress

**FIGURE 5:**
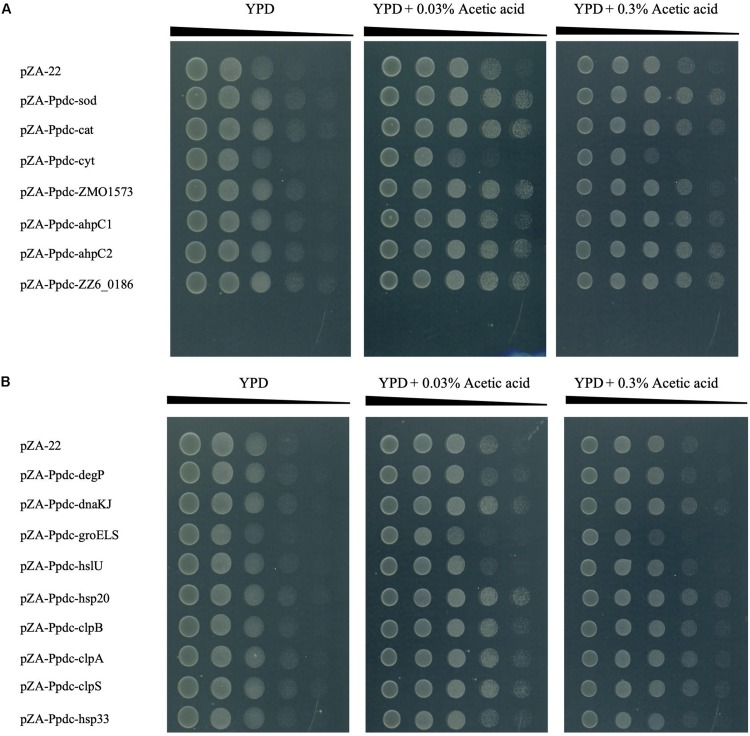
Effects of increased expression of genes for reactive oxygen species (ROS)-scavenging enzymes (RSEs) **(A)** and HSPs **(B)** on tolerance to acetic acid. Cells were grown at 30°C in YPD medium containing chloramphenicol overnight. The cell culture was serially diluted, spotted on YPD agar plates containing 0.03 or 0.3% acetic acid, and incubated at 30°C for 48 h.

The experiments described above, in which the effects of increased expression of genes for RSEs and HSPs were examined, revealed that most of the genes were effective for improvement of the CHT, accumulation of ROS, and change in cell morphology. We thus examined whether these genes allow cells to be resistant to various types of stress in addition to heat (Figure 5 and Supplementary Figure S2). When tested on plates containing different concentrations of glucose or ethanol, no transformant with any gene for RSEs and HSPs showed growth that was different from that of the control transformant. After addition of 0.03% acetic acid (about pH 5.0) and 0.3% acetic acid (about pH 4.0), all transformants including those with an empty vector grew better than those without the addition of acetic acid, which may be because the host strain has the optimal pH in the acidic range. Interestingly, transformants with some genes of RSEs, *sod*, *cat*, *ahpC1*, *ahpC2*, and *ZZ6_0186*, and transformants with some genes of HSPs, *dnaK*, *hsp20*, *clpA*, *clpB*, and *clpS*, exhibited better growth on a medium containing acetic acid, being almost consistent with their up-shift of the CHT. Therefore, it is likely that the presence of acetic acid causes the macromolecule damage by a similar mechanism to that at the CHT, but it might be more than such damage when cells are challenged by acetic acid, for example, ATP deprivation and lower pH inside the cell (Lawford and Rousseau, 1993; Ullah et al., 2013). Moreover, exogenous oxidative stress was examined by cultivation at 30°C in YPD liquid medium containing 0.1 mM H_2_O_2_. Most of the transformants with genes for RSEs and HSPs except for *degP* showed better growth than that with an empty vector (Supplementary Figure S3), suggesting that increased expression of these genes alleviated the oxidative stress by H_2_O_2_. These findings are essentially consistent with the results of measurement of ROS in these transformants, as shown in Figure 3.

## Discussion

When exposed to a CHT, mesophiles exhibit several characteristic phenotypes, including cell elongation and accumulation of ROS (Kosaka et al., 2019). To determine the CHT of mesophiles, we had developed a two-step cultivation assay (Kosaka et al., 2019), which is much clearer and more precise than a general cultivation assay that shows an increase in turbidity even at a temperature above the CHT. On the other hand, a temperature around the CHT may cause instability of the membrane, resulting in leakage of electrons to generate ROS and may give rise to unfolding or denaturing of proteins or oxidation of proteins by accumulated ROS (Jan-Ulrik et al., 2015). In this study, we thus applied the two-step cultivation method to evaluate the contribution of genes for RSEs and HSPs to the CHT of *Z. mobilis* TISTR548. Their contribution was further examined by observation of the levels of ROS accumulation and cell elongation.

Among the genes tested, enhanced expression of *sod*, *cat*, *ZMO1573*, and *ZZ6-0186* for RSEs and *dnaKJ*, *hsp20*, and *clpS* for HSPs up-shifted the CHT by 1°C, and most of the remaining genes up-shifted the CHT by 0.5–1.0°C. However, the contribution of these genes is less than the effect of thermal adaptation, which is able to increase the CHT by 2–3°C (Kosaka et al., 2019). Consistent with the weaker contribution to the up-shift of the CHT, the extents of reduction of ROS and of cell size seem to be relatively low. Nonetheless, it is likely that prevention of the accumulation of ROS is an effective way to improve the CHT.

The HSP genes tested in this study except for *degP*, *hslU*, and *hsp33* were able to raise the CHT of *Z. mobilis* when their expression was increased (Figure 2). DnaKJ and GroELS function as molecular chaperones, which are involved in refolding of unfolded or denatured proteins (Narberhaus et al., 1998; Tomoyasu et al., 1998; Thanonkeo et al., 2007; Al Refaii and Alix, 2009). Hsp20 and ClpB as Hsp100 members prevent protein aggregation and solubilize aggregated proteins, respectively (Zolkiewski et al., 2012; Mogk et al., 2015). These chaperones or Hsps deal with denaturated or aggregated proteins that are expected to appear at the CHT. ClpA and ClpS are ATP-binding subunits of ClpAP protease and its adapter, respectively (Dougan et al., 2002; Maglica et al., 2008). They may contribute to the removal of denatured or aggregated proteins at the CHT. On the other hand, increased expression of *hslU* and *hsp33* seems to inhibit cell growth even at 37.5 and 38°C. The former product is an ATP-binding subunit of HslUV protease (Yoo et al., 1998; Baytshtok et al., 2017), and the latter is a redox-regulated chaperone, which is activated by dimerization *via* disulfide bonds (Graf and Jakob, 2002). Considering these functions and activation process, the negative effects of HslU and Hsp33 on growth at the CHT might be due to proteolysis of and abnormal interaction with crucial proteins for cellular activities. In contrast to the HSP genes tested except for *hslU* and *hsp33*, *degP* for a periplasmic chaperone hardly elevated the CHT when overexpressed. A knockout mutant of *degP*, however, reduced the CHT by 1–2°C, and the gene has thus been categorized as a thermotolerant gene, which is shared by *E. coli*, *Z. mobilis*, and *A. tropicalis* (Murata et al., 2011, 2018). The inconsistency between *degP* and effective HSP genes may be due to the different cellular localization of their gene products in cells: DegP localizes in the periplasmic space, but other gene products localize in the cytoplasm. Alternatively, DegP might be sufficient to perform its activity in the parental strain. The increased expression of most of the cytoplasmic HSPs, but not DegP, may thus contribute to the maintenance of homeostasis inside cells at a CHT.

All seven of the RSE genes tested decreased the level of ROS and increased the CHT of *Z. mobilis* by their increased expression (Figures 1, 3). Four of the genes are involved in degradation of H_2_O_2_, but regulation of their expression may be distinct under different temperature conditions: the expression levels of *cytC* and *ZMO1573* are higher at 37°C than at 30°C, whereas *cat* and *ahpC* exhibit the opposite expression pattern to that of *cytC* and *ZMO1573* (Charoensuk et al., 2011). Such up-regulation of *cytC* and *ZMO1573* at a high temperature may reflect insufficient H_2_O_2_-degrading activities for survival at the CHT, being in agreement with the finding that all of the four genes are able to raise the CHT when overexpressed. On the other hand, the reduced form of thioredoxin plays an important role as an antioxidant, and its reduction requires NADPH in addition to the corresponding reductase. It is likely that NADPH is not limited in *Z. mobilis* cells at the CHT because increased expression of *ahp1* for peroxiredoxin or *ZZ6_0186* for thioredoxin reductase leads to an increase in the CHT.

This study was motivated by the surprising fact that only a few genes for RSEs and HSPs have been identified as thermotolerant genes (Charoensuk et al., 2017; Murata et al., 2018), which are essential for survival at a CHT. The finding presented suggests that many genes for RSEs and HSPs have the potential to improve the CHT, although the range of improvement is within 1°C. It is thus likely that there are several genes that have overlapping functions in cells, and thus, the disruption of one gene is complemented by another gene. In relation to this, a knockout mutant of *cytC* for a peroxidase involved in the respiratory chain exhibited filamentous shapes and reduction in growth under a shaking condition at a high temperature, and under the same condition, *sod*, *ahpC*, and *ZMO1573* are complementarily expressed to the *cytC* mutation (Charoensuk et al., 2011). Such robustness by the existence of complementing genes would have hindered the identification of genes for RSEs and HSPs as thermotolerant genes.

## Data Availability Statement

All datasets generated for this study are included in the article/Supplementary Material.

## Author Contributions

SA, MM, KK, and KS carried out the experiments. SA, MM, TK, and PT analyzed the data. MY, SA, and MM wrote the manuscript. All authors conceived this study.

## Conflict of Interest

The authors declare that the research was conducted in the absence of any commercial or financial relationships that could be construed as a potential conflict of interest.

## Supplementary Material

FIGURE S1 Checking of increased expression of genes for RSEs and HSPs from recombinant plasmids. Strains are shown by introduced gene names. Total RNA was prepared from cells cultured at 30°C as described in Materials and Methods. RT-PCR was performed with primers specific for each gene to amplify approximately 500-bp DNA fragments. After RT reaction, PCR was performed for 10, 15, 20, and 25 cycles and the products were analyzed. Each PCR product and total RNA (10 μg) as a control were subjected to 1.2% agarose gel electrophoresis and staining with ethidium bromide **(A,B)**. Intensity of stained bands was determined by using ImageJ, and the ratio of the intensity of bands from cells harboring a recombinant plasmid to that from cells harboring an empty vector, pZA22, was calculated. White columns represent the relative intensities as an expression ratio (%) **(C)**.

FIGURE S2 Effects of increased expression of genes for RSEs **(A,B)** and HSPs **(C,D)** on tolerance to various concentrations of glucose and ethanol. Cells were grown at 30°C in YPD medium containing chloramphenicol overnight. The cell culture at OD_550_ of about 1.0 was serially diluted (10^0^–10^4^-times dilution) and spotted on YPD agar plates containing 3–12% glucose or 3–5% ethanol, and incubated at 30°C for 48 h.

FIGURE S3 Effects of increased expression of genes for RSEs **(A)** and HSPs **(B)** on tolerance to H_2_O_2_. Cells were grown at 30°C in YPD medium containing 0.1 mM H_2_O_2_, and optical density at OD_550_ of the culture was measured at 12 h.

TABLE S1 Plasmids used in this study.

TABLE S2 Primers used in this study.

TABLE S3 Primers used for RT-PCR primers in this study.
