# Supplementary material for: Improvement of Thermotolerance of Zymomonas mobilis by Genes for Reactive Oxygen Species-Scavenging Enzymes and Heat Shock Proteins
Source: Front Microbiol. 2020 Jan 30;10:3073. doi: 10.3389/fmicb.2019.03073 (PMC7002363; doi:10.3389/fmicb.2019.03073)
Supplement: FIGURE S1 — Checking of increased expression of genes for RSEs and HSPs from recombinant plasmids. Strains are shown by introduced gene names. Total RNA was prepared from cells cultured at 30°C as described in Materials and Methods. RT-PCR was performed with primers specific for each gene to amplify approximately 500-bp DNA fragments. After RT reaction, PCR was performed for 10, 15, 20, and 25 cycles and the products were analyzed. Each PCR product and total RNA (10 μg) as a control were subjected to 1.2% agarose gel electrophoresis and staining with ethidium bromide (A,B). Intensity of stained bands was determined by using ImageJ, and the ratio of the intensity of bands from cells harboring a recombinant plasmid to that from cells harboring an empty vector, pZA22, was calculated. White columns represent the relative intensities as an expression ratio (%) (C). [file Presentation_1.PPTX]

## Slide 1
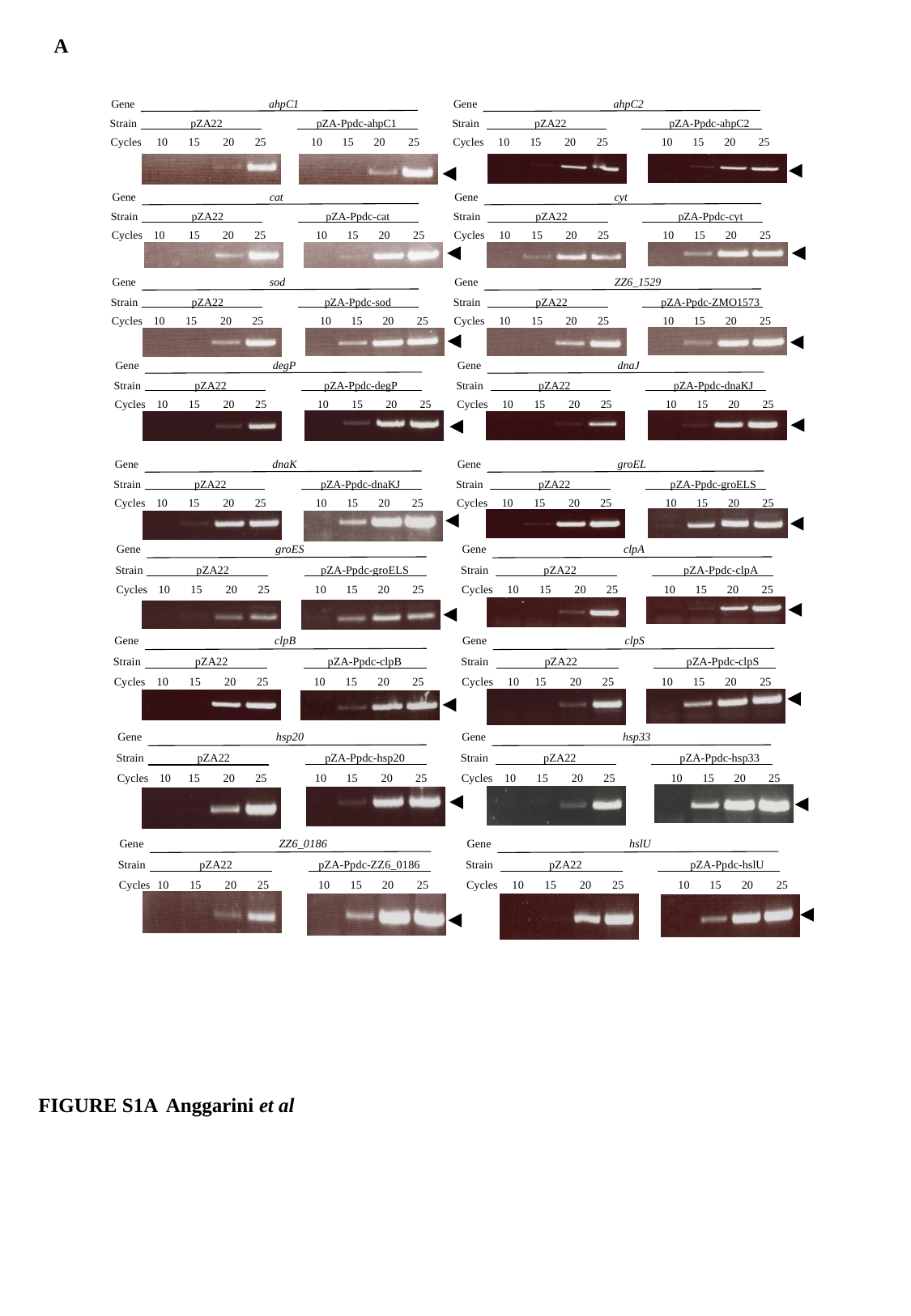

A
Gene
ahpC1
Gene
ahpC2
Strain
pZA22
pZA-Ppdc-ahpC1
Strain
pZA22
pZA-Ppdc-ahpC2
Cycles
10
15
20
25
10
15
20
25
Cycles
10
15
20
25
10
15
20
25
Gene
cat
Gene
cyt
Strain
pZA22
pZA-Ppdc-cat
Strain
pZA22
pZA-Ppdc-cyt
Cycles
10
15
20
25
10
15
20
25
Cycles
10
15
20
25
10
15
20
25
Gene
sod
Gene
ZZ6_1529
Strain
pZA22
pZA-Ppdc-sod
Strain
pZA22
pZA-Ppdc-ZMO1573
Cycles
10
15
20
25
10
15
20
25
Cycles
10
15
20
25
10
15
20
25
Gene
degP
Gene
dnaJ
Strain
pZA22
pZA-Ppdc-degP
Strain
pZA22
pZA-Ppdc-dnaKJ
Cycles
10
15
20
25
10
15
20
25
Cycles
10
15
20
25
10
15
20
25
Gene
dnaK
Gene
groEL
Strain
pZA22
pZA-Ppdc-dnaKJ
Strain
pZA22
pZA-Ppdc-groELS
Cycles
10
15
20
25
10
15
20
25
Cycles
10
15
20
25
10
15
20
25
Gene
groES
Gene
clpA
Strain
pZA22
pZA-Ppdc-groELS
Strain
pZA22
pZA-Ppdc-clpA
Cycles
10
15
20
25
10
15
20
25
Cycles
10
15
20
25
10
15
20
25
Gene
clpB
Gene
clpS
Strain
pZA22
pZA-Ppdc-clpB
Strain
pZA22
pZA-Ppdc-clpS
Cycles
10
15
20
25
10
15
20
25
Cycles
10
15
20
25
10
15
20
25
Gene
hsp20
Gene
hsp33
Strain
pZA22
pZA-Ppdc-hsp20
Strain
pZA22
pZA-Ppdc-hsp33
Cycles
10
15
20
25
10
15
20
25
Cycles
10
15
20
25
10
15
20
25
Gene
ZZ6_0186
Gene
hslU
Strain
pZA22
pZA-Ppdc-ZZ6_0186
Strain
pZA22
pZA-Ppdc-hslU
Cycles
10
15
20
25
10
15
20
25
Cycles
10
15
20
25
10
15
20
25
FIGURE S1A Anggarini et al

## Slide 2
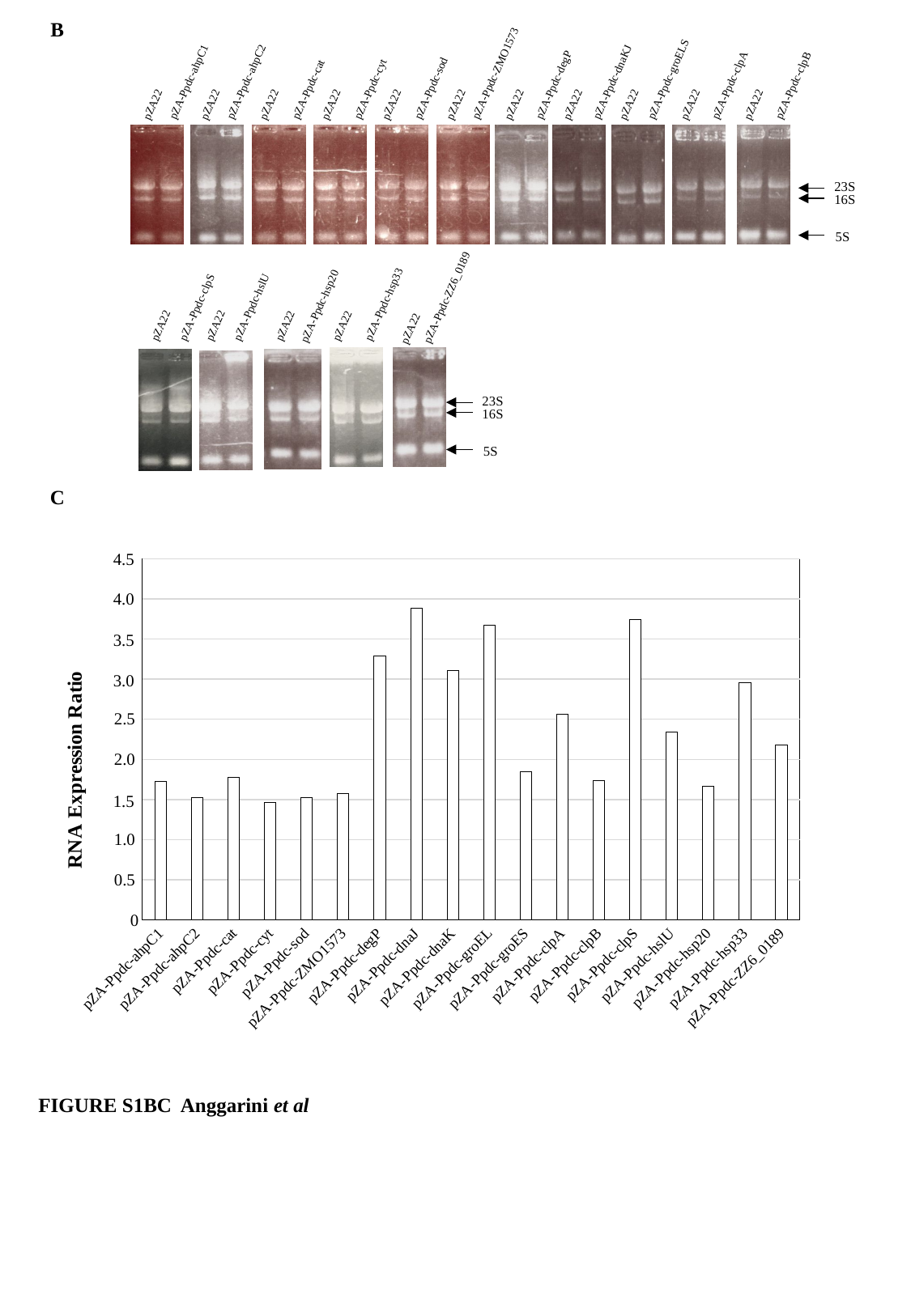

B
pZA-Ppdc-ZMO1573
pZA-Ppdc-groELS
pZA-Ppdc-ahpC1
pZA-Ppdc-ahpC2
pZA-Ppdc-dnaKJ
pZA-Ppdc-degP
pZA-Ppdc-clpA
pZA-Ppdc-clpB
pZA-Ppdc-sod
pZA-Ppdc-cyt
pZA-Ppdc-cat
pZA22
pZA22
pZA22
pZA22
pZA22
pZA22
pZA22
pZA22
pZA22
pZA22
pZA22
23S
16S
5S
pZA-Ppdc-ZZ6_0189
pZA-Ppdc-hsp33
pZA-Ppdc-hsp20
pZA-Ppdc-hslU
pZA-Ppdc-clpS
pZA22
pZA22
pZA22
pZA22
pZA22
23S
16S
5S
C
### Chart
| Category | |
|---|---|
| pZA-Ppdc-ahpC1 | 1.727838345337855 |
| pZA-Ppdc-ahpC2 | 1.52167362870643 |
| pZA-Ppdc-cat | 1.777945304815445 |
| pZA-Ppdc-cyt | 1.467117236358823 |
| pZA-Ppdc-sod | 1.520207317197348 |
| pZA-Ppdc-ZMO1573 | 1.574540333776104 |
| pZA-Ppdc-degP | 3.292255892255892 |
| pZA-Ppdc-dnaJ | 3.887085291186103 |
| pZA-Ppdc-dnaK | 3.111642807087796 |
| pZA-Ppdc-groEL | 3.674072304507087 |
| pZA-Ppdc-groES | 1.85076000076671 |
| pZA-Ppdc-clpA | 2.563304923430452 |
| pZA-Ppdc-clpB | 1.73990080076491 |
| pZA-Ppdc-clpS | 3.743936112615051 |
| pZA-Ppdc-hslU | 2.344446817599316 |
| pZA-Ppdc-hsp20 | 1.660595518264607 |
| pZA-Ppdc-hsp33 | 2.950614470293297 |
| pZA-Ppdc-ZZ6_0189 | 2.175923376755104 |4.5
4.0
3.5
3.0
2.5
2.0
1.5
1.0
0.5
 0
FIGURE S1BC Anggarini et al

## Slide 3
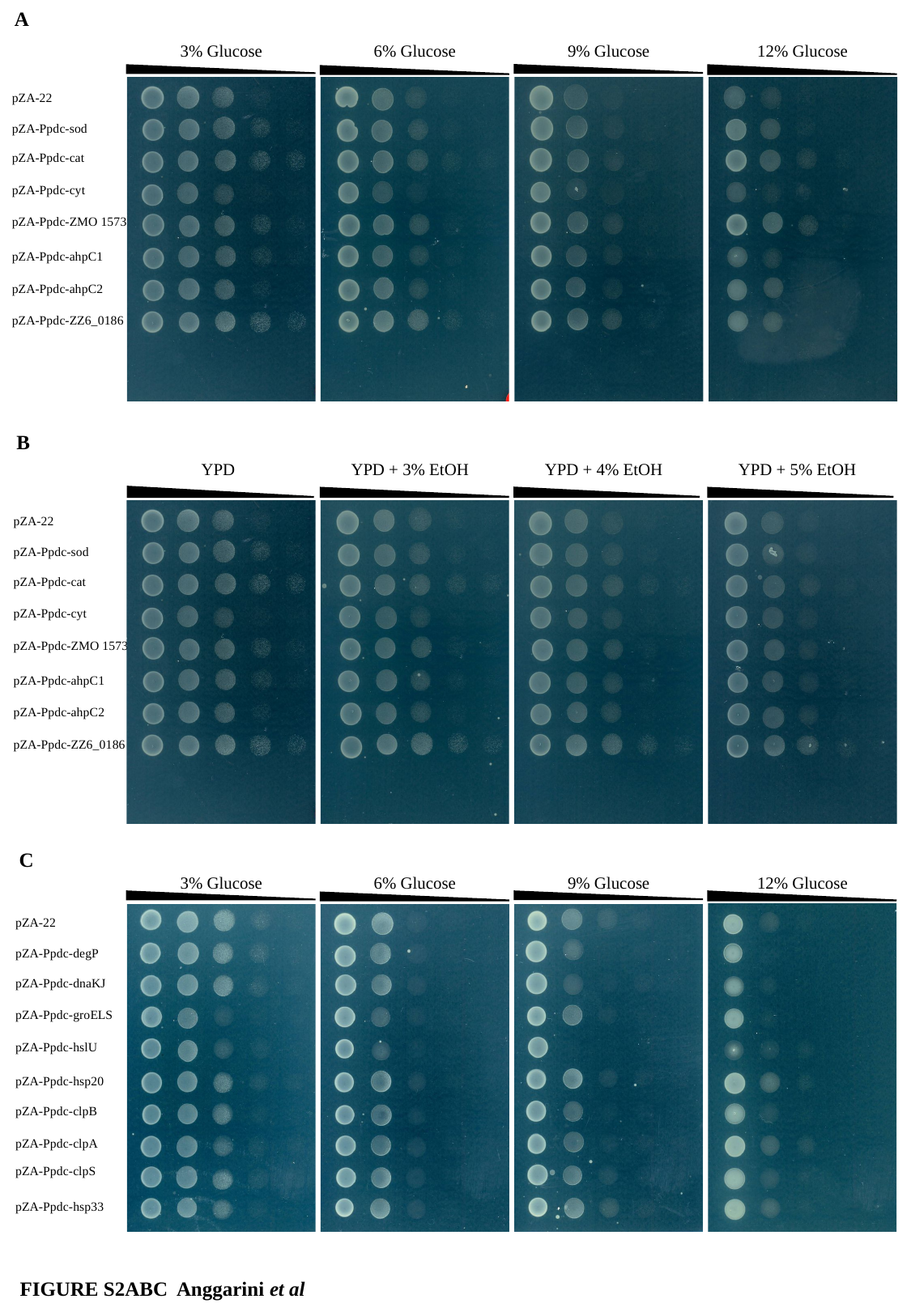

A
6% Glucose
9% Glucose
12% Glucose
3% Glucose
pZA-22
pZA-Ppdc-sod
pZA-Ppdc-cat
pZA-Ppdc-cyt
pZA-Ppdc-ZMO 1573
pZA-Ppdc-ahpC1
pZA-Ppdc-ahpC2
pZA-Ppdc-ZZ6_0186
B
 YPD
YPD + 3% EtOH
YPD + 4% EtOH
YPD + 5% EtOH
pZA-22
pZA-Ppdc-sod
pZA-Ppdc-cat
pZA-Ppdc-cyt
pZA-Ppdc-ZMO 1573
pZA-Ppdc-ahpC1
pZA-Ppdc-ahpC2
pZA-Ppdc-ZZ6_0186
C
6% Glucose
9% Glucose
12% Glucose
3% Glucose
pZA-22
pZA-Ppdc-degP
pZA-Ppdc-dnaKJ
pZA-Ppdc-groELS
pZA-Ppdc-hslU
pZA-Ppdc-hsp20
pZA-Ppdc-clpB
pZA-Ppdc-clpA
pZA-Ppdc-clpS
pZA-Ppdc-hsp33
FIGURE S2ABC Anggarini et al

## Slide 4
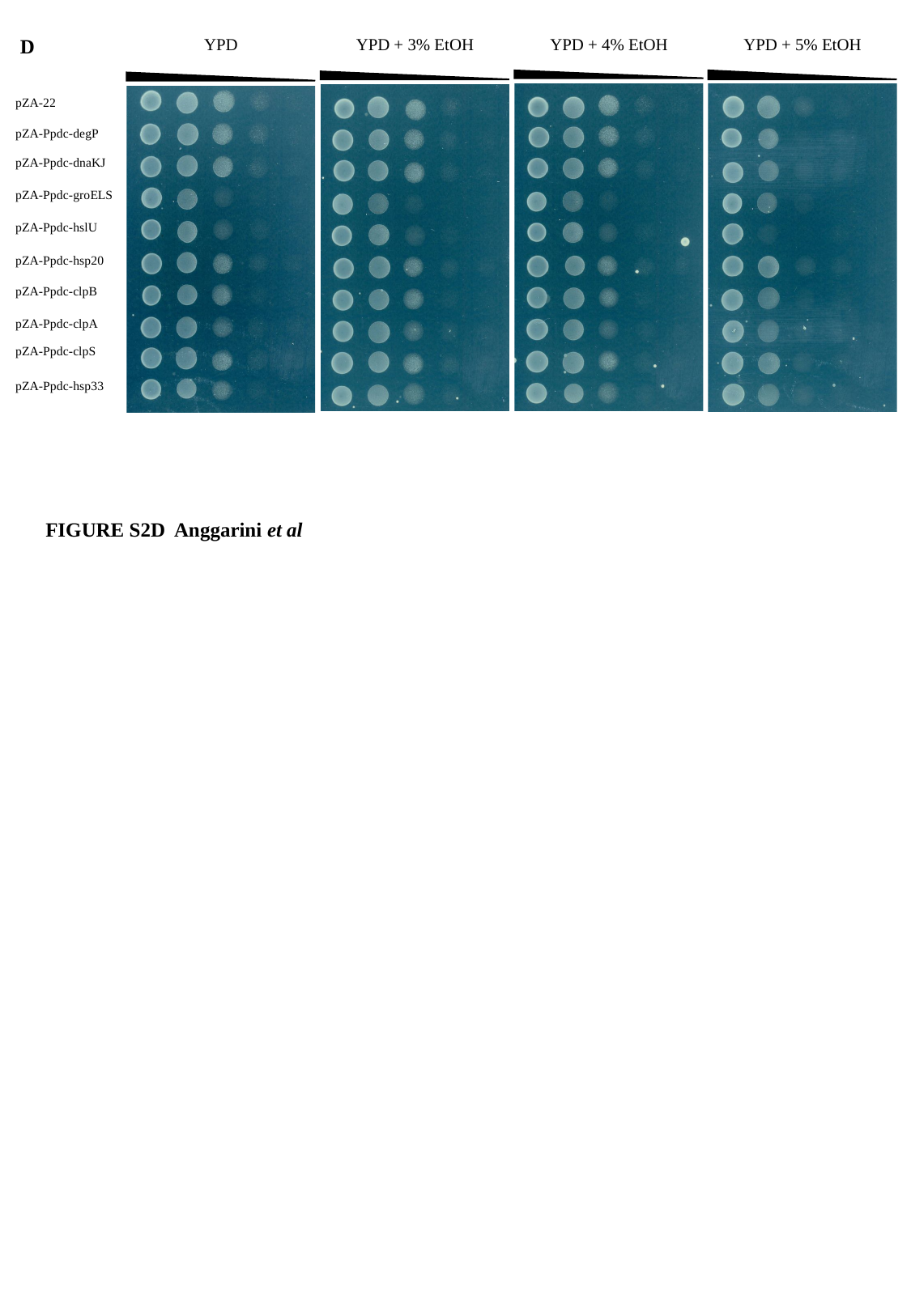

YPD
YPD + 3% EtOH
YPD + 4% EtOH
YPD + 5% EtOH
D
pZA-22
pZA-Ppdc-degP
pZA-Ppdc-dnaKJ
pZA-Ppdc-groELS
pZA-Ppdc-hslU
pZA-Ppdc-hsp20
pZA-Ppdc-clpB
pZA-Ppdc-clpA
pZA-Ppdc-clpS
pZA-Ppdc-hsp33
FIGURE S2D Anggarini et al

## Slide 5
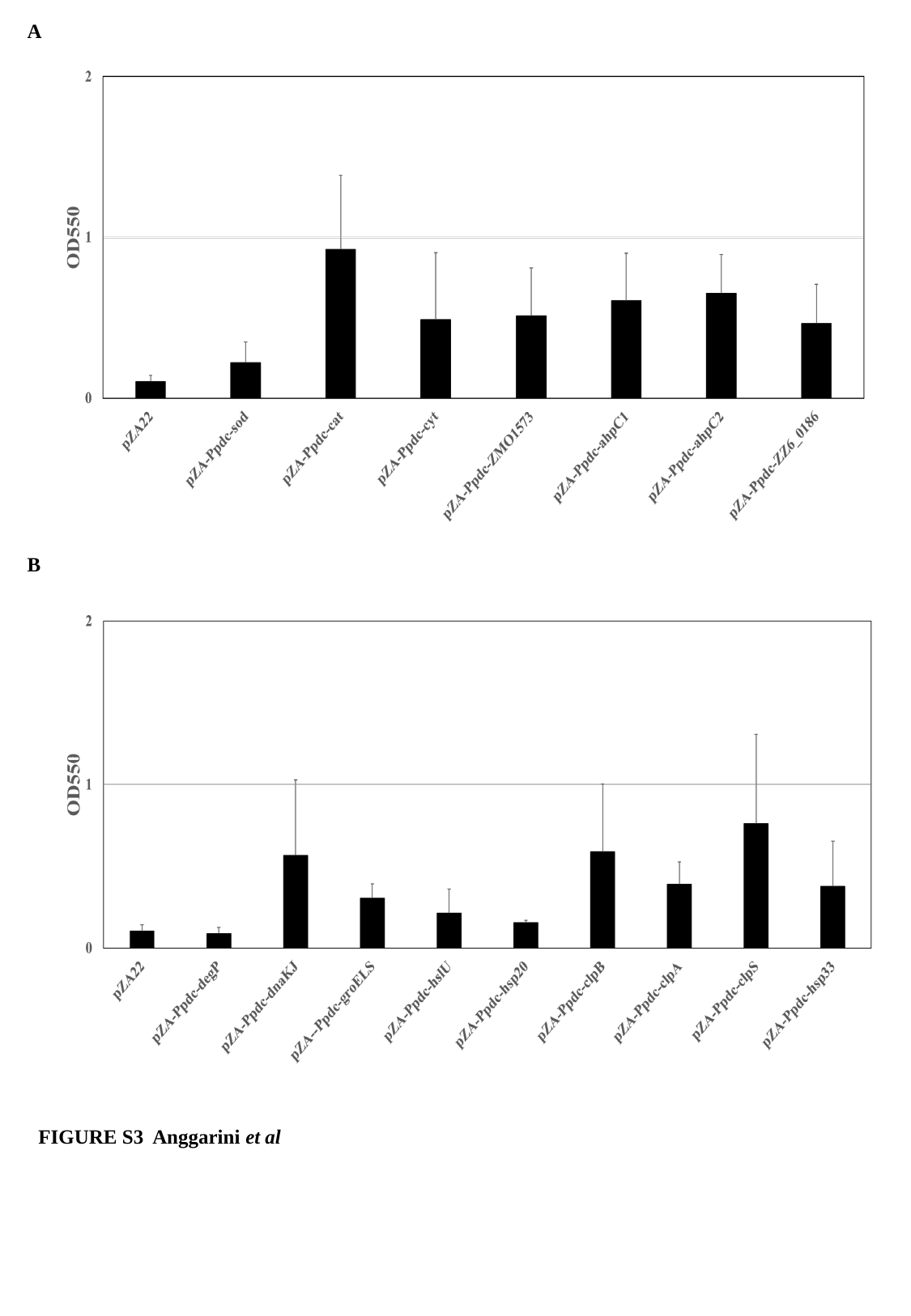

A
B
FIGURE S3 Anggarini et al

## Slide 6
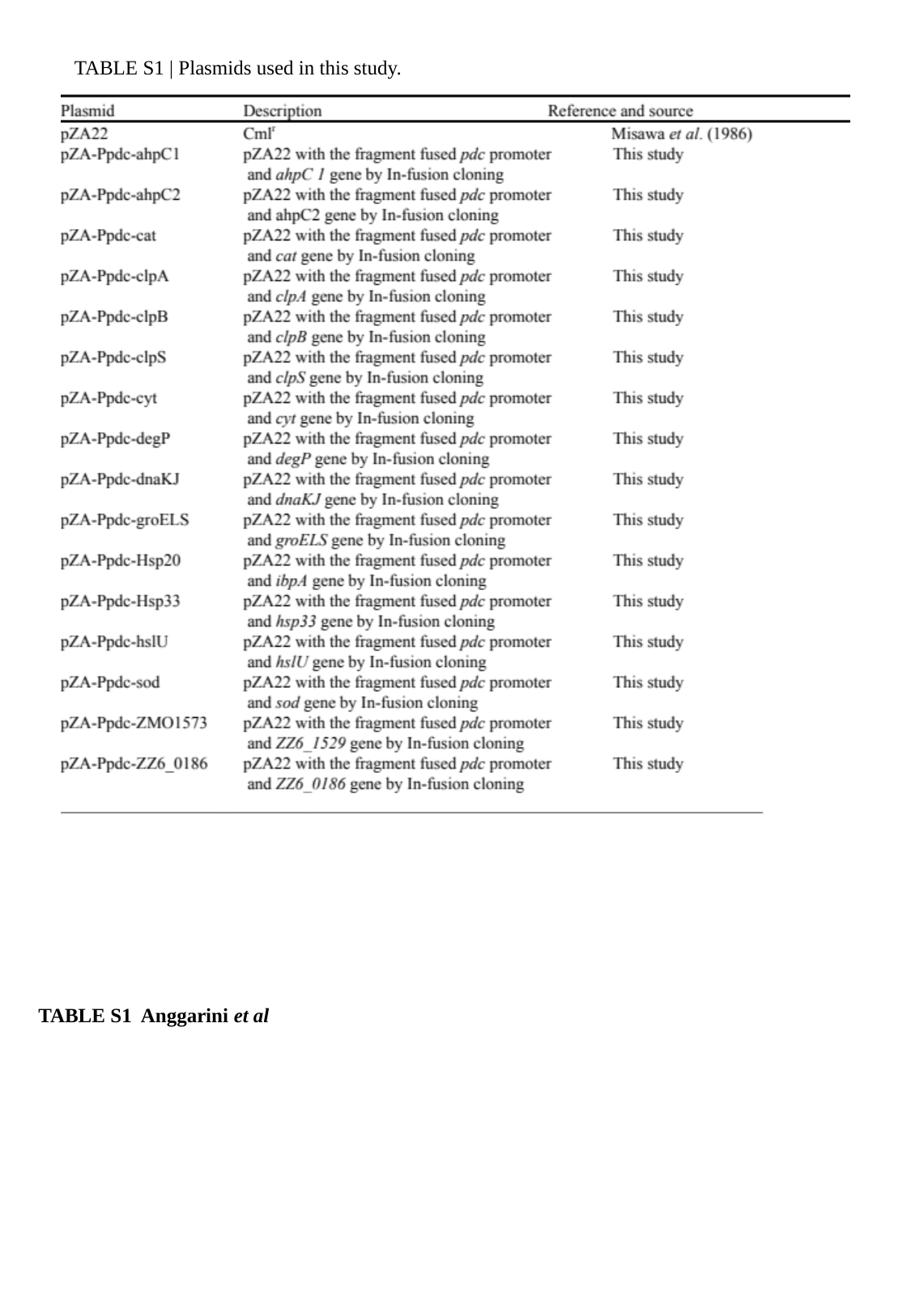

TABLE S1 | Plasmids used in this study.
TABLE S1 Anggarini et al

## Slide 7
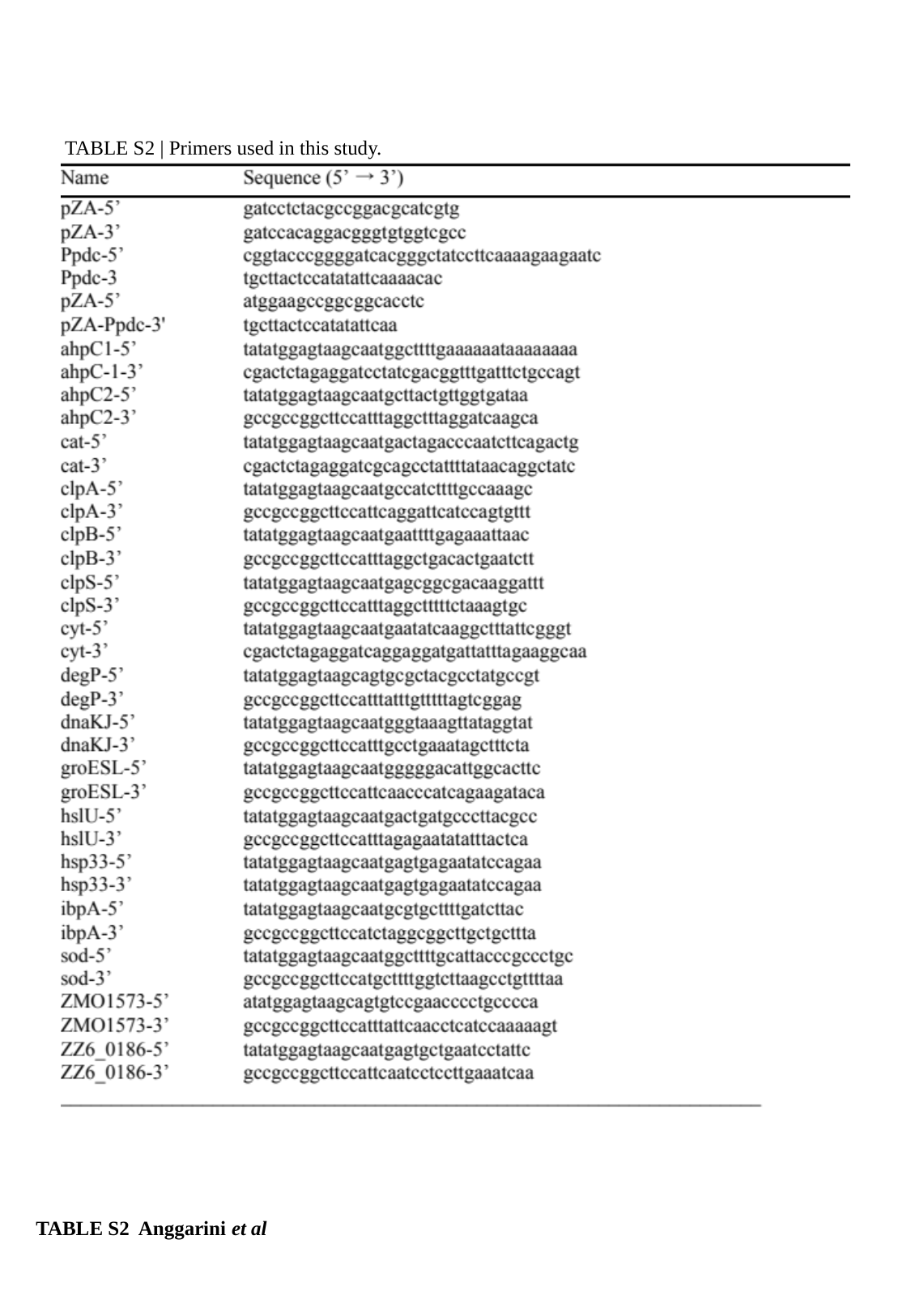

TABLE S2 | Primers used in this study.
TABLE S2 Anggarini et al

## Slide 8
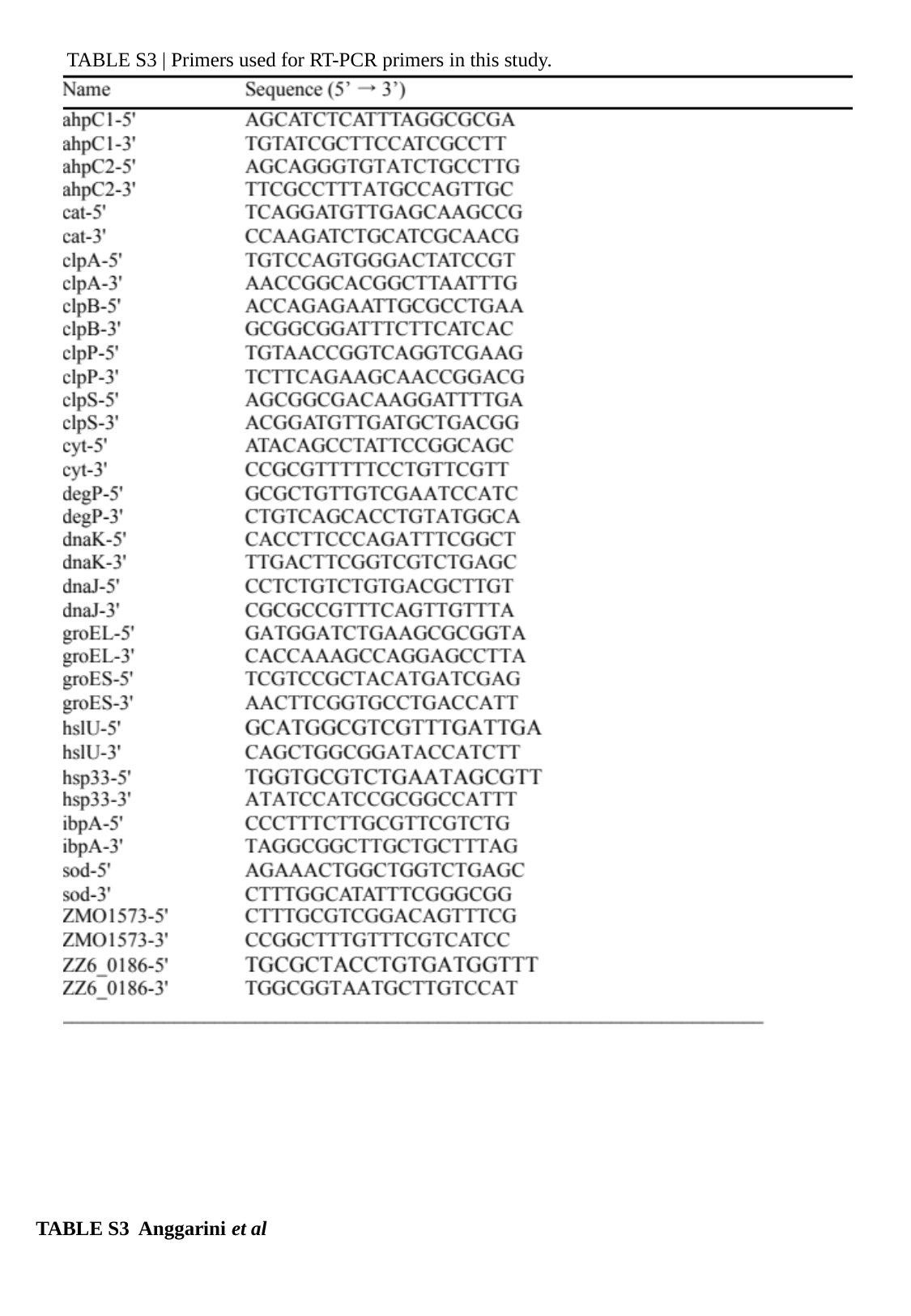

TABLE S3 | Primers used for RT-PCR primers in this study.
TABLE S3 Anggarini et al
